# Short Report: Adult *Aedes* abundance and risk of dengue transmission

**DOI:** 10.1371/journal.pntd.0009475

**Published:** 2021-06-03

**Authors:** Janet Ong, Joel Aik, Lee Ching Ng

**Affiliations:** 1 Environmental Health Institute, National Environment Agency, Singapore, Singapore; 2 School of Biological Sciences, Nanyang Technological University, Singapore, Singapore; Faculty of Science, Mahidol University, THAILAND

## Abstract

Dengue is transmitted mainly by the adult female *Aedes aegypti* mosquito. However, little is known about the impact of adult *Aedes* abundance on the risk of dengue transmission. Here we analysed nationally representative dengue case and vector surveillance data collected from Singapore, to determine the effect of adult *Aedes* abundance on the risk of dengue transmission. A case was an area with active dengue transmission as indicated by the presence of dengue cluster. A control was an area where no dengue cluster was reported. Using multivariate logistic regression, we analysed 88 cases and 602 controls and estimated the odds of dengue cluster formation at various adult *Aedes* abundance levels, estimated by the mean number of adult female *Aedes* per Gravitrap per week and categorised into Low, Moderate, High and Very High abundance level. We found that the risk of dengue cluster formation was positively associated with adult *Ae*. *aegypti* abundance. We observed a three to four-fold increase in the odds of dengue clusters forming in areas with High (AOR: 3.40, 95% CI: 2.09, 5.52) and Very High (AOR: 3.99, 95% CI: 2.46, 6.46) adult *Aedes aegypti* abundance level compared to those with low *Ae*. *aegypti* abundance level. Our study strengthens the evidence for the use of adult *Aedes* indices for dengue risk assessment and early warning for dengue outbreaks. Entomological indicators of adult *Ae*. *aegypti* could be used to anticipate and prioritize areas for dengue control.

## Author summary

The House Index and Breteau index are the most widely used indices for vector surveillance. However, the appropriateness of these larval indices has been questioned as they have not been satisfactorily linked to disease transmission. Indices based on actual counts of adult *Aedes* are likely to be more useful in assessing transmission risk, but the adult *Aedes* population is rarely sampled as such sampling is perceived as time-consuming and difficult. In this study, which used national data from Singapore, we investigated the effect of adult *Aedes* abundance on the risk of dengue transmission. We found that areas with high *Ae*. *aegypti* abundance had a higher odds of dengue cluster formation. Our study findings suggest that the adult *Ae*. *aegypti* abundance can allow early identification of geographical localities at high risk of dengue transmission and thus, a useful indicator to anticipate and prioritize areas for dengue control.

## Introduction

Dengue is currently regarded as one of the most important mosquito-borne viral diseases. The global incidence of dengue has been increasing drastically around the world in recent decades [1,2]. One study estimates that 390 million dengue infections occur worldwide each year, of which 96 million manifest clinically [3]. Dengue is endemic throughout the tropics, and almost half of the world’s population are at risk of infection, the majority of whom (70%) live in the Asia-Pacific region [1]. The geographical range of dengue is expected to further expand due to climate change and urbanisation [4].

Dengue is transmitted primarily by the vector *Aedes aegypti*, and a secondary vector *Aedes albopictus* [5]. Vector surveillance, recommended by the World Health Organization, is a routine practice in many dengue endemic countries to provide quantifiable measure of fluctuations in magnitude and geographical distribution of dengue vector populations [6]. The House Index and Breteau Index are the most widely used indices for vector surveillance [1,6]. However, the appropriateness of these indices had been questioned as they have not been satisfactorily linked to disease transmission [7,8]. These larval indices do not seem to reliably assess transmission risk, which policy makers typically rely on to define thresholds for dengue epidemic alerts and to set targets for vector control programs [9,10]. Indices based on actual counts of adult *Ae*. *aegypti* are likely to be more useful in assessing transmission risk, but the adult *Ae*. *aegypti* population is rarely sampled as such sampling is perceived as time-consuming and difficult [6,11]. Another difficulty for adult sampling is the variability linked to the adult sampling tools since each sampling tool has its own attractiveness and can provide very different numbers when used in the same location [12]. Thus, only a few studies have attempted to examine the relationship between the adult *Ae*. *aegypti* and dengue transmission [8]. This study therefore aimed to assess the effect of adult *Aedes* abundance on risk of dengue transmission using nationally representative surveillance data in Singapore.

## Methods

### Ethics statement

The study was granted approval by the Environmental Health Institute of the National Environment Agency, Singapore. The study did not involve human participants.

### Study setting

Located within Southeast-Asia, Singapore is a city-state with a land area of 724.2km^2^ and an estimated population of 5.8 million *[13]*. Singapore experiences a tropical climate, characterized by abundant rainfall, high and uniform temperatures and high humidity throughout the year *[14]*. Dengue is hyper-endemic in this country with year-round circulation of all four dengue virus (DENV) serotypes and a cyclic epidemic pattern that oscillates between DENV-1 and DENV-2 as the dominant serotype *[15]*. In 2019 and 2020, Singapore experienced explosive dengue outbreaks, reporting 15,998 and 35,315 laboratory confirmed dengue cases respectively [16,17]. The low herd immunity levels of Singapore’s resident population and the increased human population density are factors that may have contributed to the population’s susceptibility to outbreaks [18,19].

There are three types of residences in Singapore: public high-rise apartment blocks, private high-rise apartment blocks and landed houses. Built by the government, the public high-rise apartment blocks are the most common housing type, accounting for close to 80% of Singapore residence [20]. These apartment blocks range from three to 50 storeys, with the majority between 12 and 30 storeys. The remaining residents live in private high-rise apartment blocks and landed houses built by private developers.

### Entomological data

**Fig 1:**
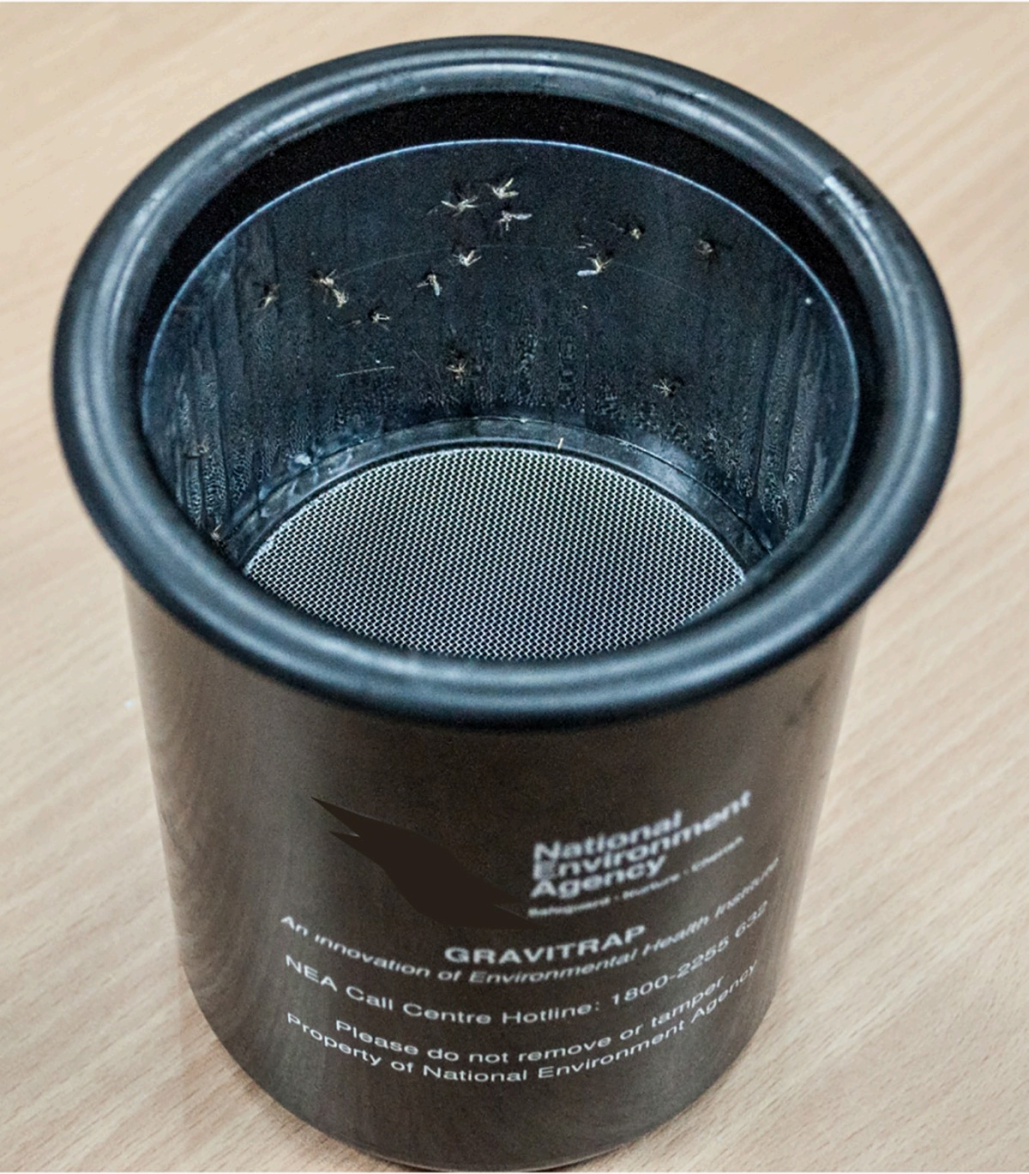
Photo of a Gravitrap with mosquitoes trapped on the sticky lining.

Gravitraps (Fig 1), which lure and trap gravid female *Aedes* mosquitoes, were used as a surveillance tool by the National Environment Agency (NEA), the lead government authority for vector control in Singapore. NEA has deployed Gravitraps across all public residential high-rise apartment blocks, to monitor the spatio-temporal variability of the adult *Aedes* population. Gravitraps were placed along the common corridors of the apartment blocks in the ratio of 1 Gravitrap for every 20 households (i.e. two Gravitraps on each of three floors per apartment block: lower floor(2^nd^), mid floor (5^th^ or 6^th^) and high floor (10^th^ or 11^th^). Mosquitoes were collected from the Gravitraps on a weekly basis and are entomologically identified in the laboratory using mosquito identification keys [21,22]. More than 50,000 Gravitraps were deployed as of 2019 [23].

### Dengue case data

In Singapore, the reporting of all laboratory confirmed dengue infections is mandatory. These reports are consolidated through the national surveillance system by the Ministry of Health (Singapore) and transmitted to the NEA for the purpose of implementing disease control activities. Reported dengue infections are linked in space and time in order to determine areas of elevated dengue transmission requiring control activities such as house-to-house inspections to search and destroy mosquito breeding habitats and community engagement to create awareness for dengue and eliciting dengue prevention action [24]. Such areas are defined as “dengue clusters” and each area is formed when two or more reported infections fall within a 150-meter radius and with illness onset dates within a 14-day period of each other [25].

### Statistical analysis

**Fig 2:**
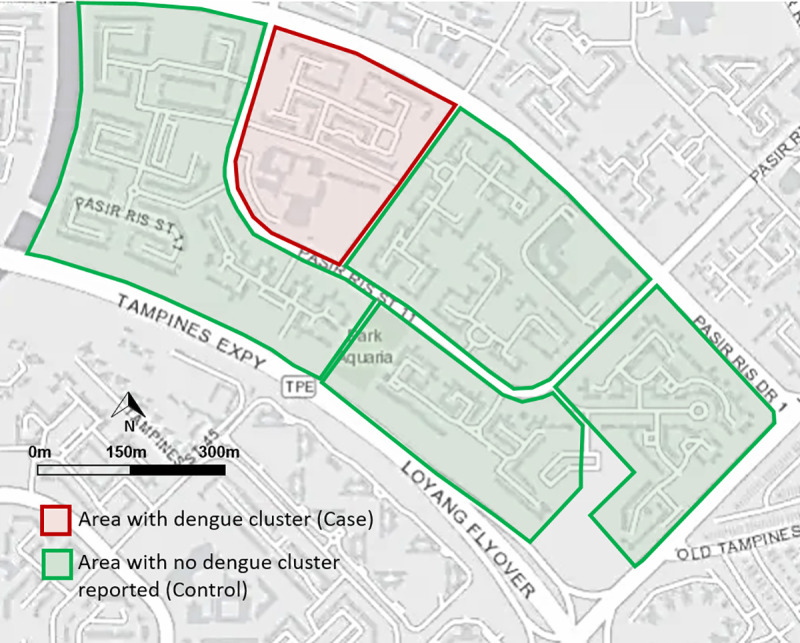
Map showing an example of a case (red) and controls (green). The figure was created with base layer obtained from https://landsatlook.usgs.gov/.

**Fig 3:**
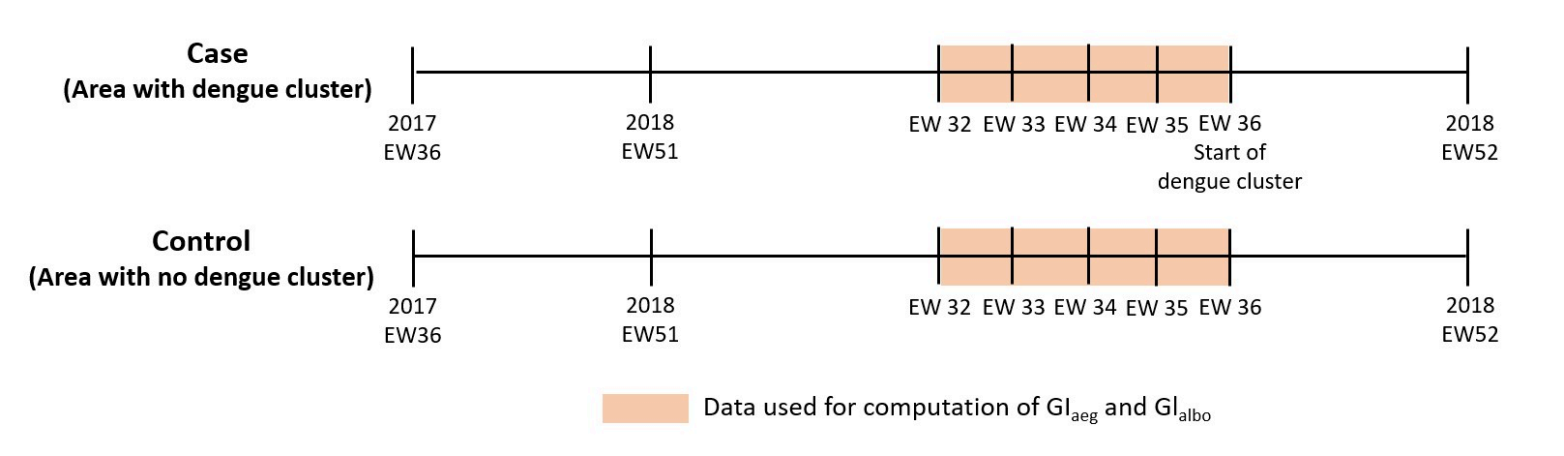
Illustration on the computation of mean GI_aeg_ and GI_albo_ in cases (area with dengue cluster) and controls (area with no dengue cluster).

We obtained records of all dengue clusters and adult *Aedes* vector data between September 2017 and December 2018. We restricted our analysis to areas deployed with Gravitraps. We used an epidemiological “case-control” study design to examine the effect of adult *Aedes* abundance on the risk of dengue transmission. We defined a case as an area with a dengue cluster and a control as an area (i.e. a group of 10–15 public high-rise apartment blocks that are typically 150m apart and bounded by the roads) where no dengue cluster was reported throughout the study duration (Fig 2). The mean *Ae*. *aegypti* trap rate (GI_aeg_) and mean *Ae*. *albopictus* trap rate (GI_albo_), defined as the mean number of adult female *Ae*. *aegypti* and *Ae*. *albopictus* trapped per Gravitrap per week respectively, were used to estimate the adult *Aedes* abundance in an area. We calculated the mean GI_aeg_ and GI_albo_ of each dengue cluster, four weeks preceding its formation. Similarly, we calculated the mean GI_aeg_ and GI_albo_ from the same four-week period for the controls (Fig 3). Wilcoxon rank-sum test was used to test for differences in the GI_aeg_ and GI_albo_ between the two groups. To assess the association between adult *Aedes* abundance and risk of dengue transmission, we stratified the GI_aeg_ and GI_albo_ into four levels (i.e. Low, Moderate, High and Very High) based on quartiles. We estimated the odds of dengue cluster formation at different *Aedes* abundance levels by fitting a multivariable logistic regression with the outcome measure being the presence of dengue clusters (cases) coded as “1” and the absence of dengue clusters (controls) as “0”, the GI_aeg_ and GI_albo_ as the independent variable, and adjusting for seasonal effect and potential confounder such as the geographical districts. Due to data unavailability, we were not able to account for the effect of other confounders such as the localized initiatives for dengue prevention and control.

## Results

**Fig 4:**
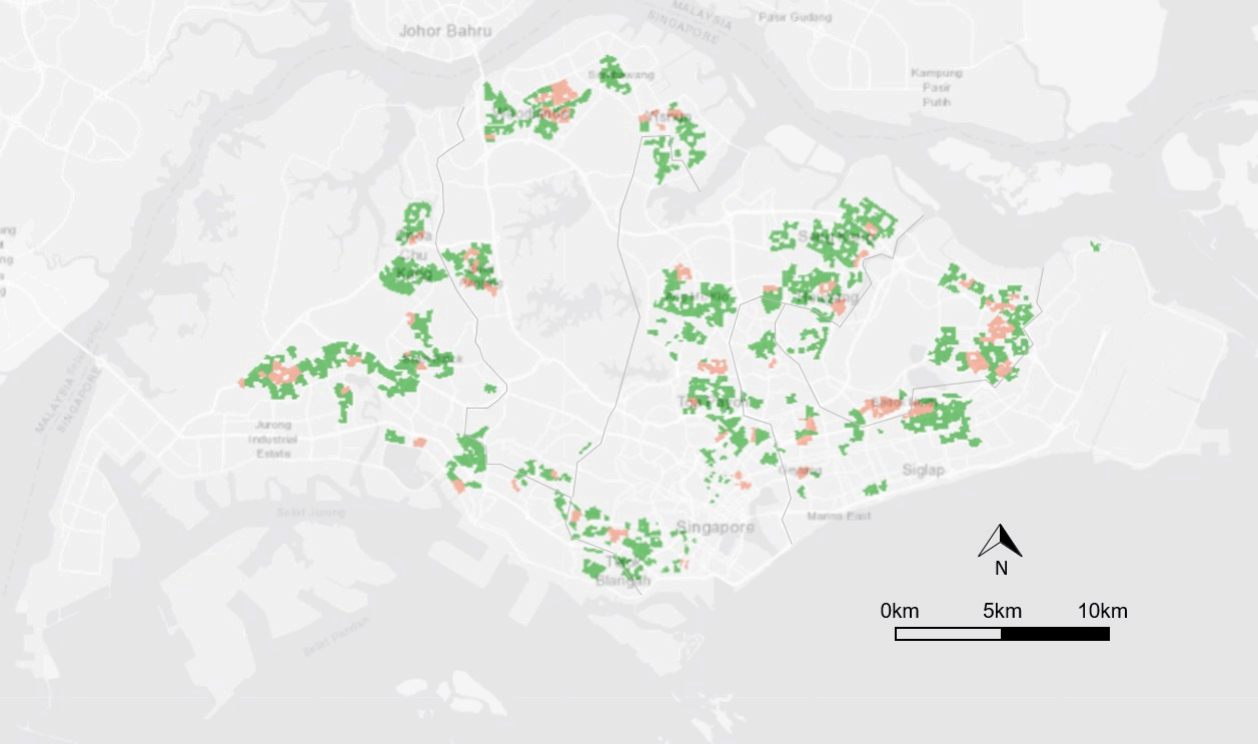
Map showing the spatial distribution of the cases (red) and controls (green). **The grey boundary lines indicate the five geographical districts in Singapore. The figure was created using R software with base layer obtained from https://landsatlook.usgs.gov/**.

**Fig 5:**
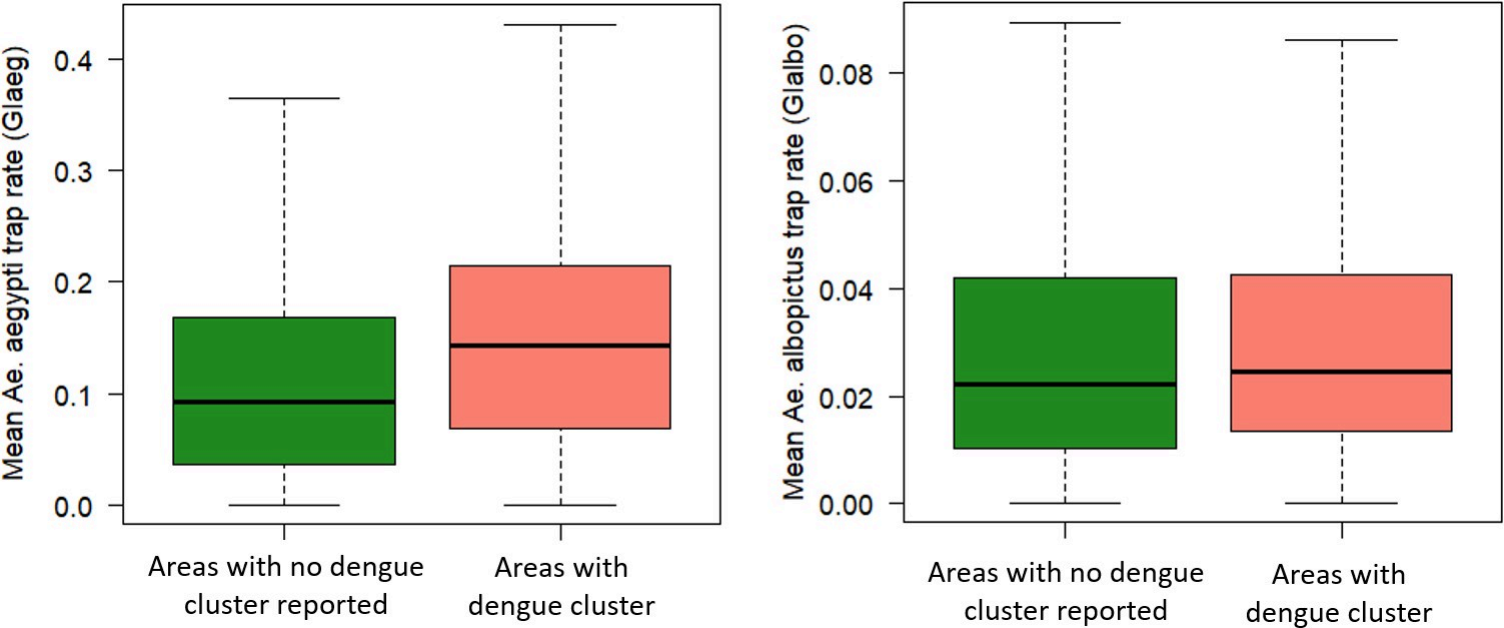
Comparison of the GI_aeg_ and GI_albo_ between cases (areas with dengue cluster) and controls (areas with no dengue cluster reported).

Over the study duration of 69 weeks, the GI_aeg_ ranged between 0.00 and 0.98 and averaging at 0.12 while the GI_albo_ ranged between 0.00 to 0.79 and averaging at 0.03. There were small variations in the GI_aeg_ and GI_albo_ throughout the year (S1 Fig). The GI_aeg_ and GI_albo_ distributions were generally the same across the five geographical districts except the South-East district, which had a lower GI_albo_ (Tables 1 and 2)_._ We analysed 88 cases and 602 controls. Fig 4 shows the spatial distribution of the cases and controls. The cases and controls are well distributed across the country and thus nationally representative. Areas with dengue cluster had a higher GI_aeg_ than those with no dengue cluster. There was no significant difference in the Gl_albo_ between the two groups (Fig 5).

**Table 1:** Summary statistics of the mean *Ae*. *aegypti* trap rates, GI_aeg_. Interquartile range refers to the 1^st^ and 3^rd^ quartile.

Variable	Mean (SD)	Median	Interquartile Range	Minimum	Maximum
**Overall**	0.12 (0.09)	0.10	0.05–0.17	0.00	0.98
**Geographical districts**					
Central	0.11 (0.09)	0.10	0.05–0.16	0.00	0.91
North-East	0.13 (0.12)	0.11	0.04–0.19	0.00	0.81
North-West	0.11 (0.10)	0.09	0.04–0.16	0.00	0.74
South-East	0.11 (0.08)	0.10	0.05–0.16	0.00	0.53
South-West	0.12 (0.11)	0.10	0.04–0.17	0.00	0.98

**Table 2:** Summary statistics of the mean *Ae*. *albopictus* trap rates, GI_albo_. Interquartile range refers to the 1^st^ and 3^rd^ quartile.

Variable	Mean (SD)	Median	Interquartile Range	Minimum	Maximum
**Overall**	0.03 (0.03)	0.02	0.01–0.04	0.00	0.79
**Geographical districts**					
Central	0.04 (0.03)	0.03	0.01–0.05	0.00	0.29
North-East	0.03 (0.02)	0.02	0.01–0.04	0.00	0.22
North-West	0.03 (0.03)	0.02	0.01–0.04	0.00	0.24
South-East	0.02 (0.04)	0.01	0.005–0.03	0.00	0.79
South-West	0.04 (0.03)	0.03	0.01–0.05	0.00	0.41

For the univariate analysis, the odds of dengue cluster forming in areas with Moderate, High and Very High GI_aeg_ level was 2.22 (95% CI: 1.37, 3.60), 3.30 (95% CI: 2.07, 5.27) and 4.14 (95% CI: 2.63, 6.49) compared to that of areas with Low GI_aeg_ (S1 Table). After adjusting for the effects of potential confounders, the risk of dengue transmission in areas with Moderate, High and Very High GI_aeg_ level remained high. We observed a two to four-fold increase in the odds of dengue clusters in areas with Moderate GI_aeg_ (AOR: 2.38, 95% CI: 1.45, 3.89), High GI_aeg_ (AOR: 3.40, 95% CI: 2.09, 5.52) and Very High GI_aeg_ (AOR: 3.99, 95% CI: 2.46, 6,46) compared to those with Low GI_aeg_ (Table 3). There was no significant difference in the odds of dengue cluster among the different GI_albo_ levels (S2 Table). Among the geographical districts, North-East district (AOR: 2.95, 95% CI: 2.02, 4.31) had the highest odds of dengue cluster formation compared to the Central district. Among the epidemiological month, March, April, July and August had lower odds of dengue cluster formation relative to January (S2 Table).

**Table 3:** Adjusted odds ratios for factors associated with dengue cluster formation.

Variable		Odds Ratio	95% CI
**Mean *Ae*. *aegypti* trap rate (GI**_**aeg**_**)**			
Low:	GI_aeg_ < 0.05	Referent	
Moderate:	0.05 ≤ GI_aeg_ < 0.10	2.38	1.45–3.89
High:	0.10 ≤ GI_aeg_ < 0.17	3.40	2.09–5.52
Very High:	GI_aeg_ ≥ 0.17	3.99	2.46–6.46
**Geographical district**			
Central		Referent	
North-East		2.95	2.02–4.31
North-West		1.81	1.20–2.72
South-East		1.10	0.59–2.03
South-West		1.64	1.06–2.53

## Discussion

Various researchers have investigated the relationship between dengue transmission and the immature stages of *Aedes—*larval and pupal [26–28]. However, studies examining the relationship between dengue transmission and the adult *Aedes* remain scarce. In this present study, we examined the effect of adult *Aedes* abundance on risk of dengue transmission. After adjusting for the effect of potential confounders, we found that the risk of dengue transmission was positively associated with adult *Ae*. *aegypti* abundance. This finding was consistent with the few studies in existing literature. A study in Venezuela found a positive correlation between the number of dengue cases and abundance of *Ae*. *aegypti* adults [29]. Another study in Puerto Rico also showed that the number of adult *Ae*. *aegypti* was a significant risk factor for dengue infection [30]. Our study findings suggest that the entomological measures of adult *Ae*. *aegypti* can be a useful indicator to anticipate and prioritize areas for dengue control.

In our study, the adult *Ae*. *albopictus* abundance was not a significant predictor of dengue transmission. This is not surprising since *Ae*. *albopictus* is native and ubiquitous throughout Singapore [31]. Moreover, *Ae*. *albopictus* is generally known to be a less efficient vector of dengue than *Ae*. *aegypti* as it is not as well adapted to urban environments and is less anthropophilic than *Ae*. *aegypti* [32]. There were spatial differences in the transmission risk among the geographical districts of residence, with higher levels observed in the North-East district compared to the others. This appears to correspond with historical trends for dengue reports in Singapore which showed that North-East district had comparatively higher levels of reported dengue [31].

To the best of our knowledge, this is the first large scale, nationally representative study that examined the effect of adult *Aedes* abundance on risk of dengue transmission. Unlike previous studies, we used a case-control study design whereby entomological data were collected from areas of confirmed dengue cluster as well as control areas (i.e. no dengue cluster). We only used laboratory confirmed dengue infections, and thus the potential for case misclassification was low. Although unreported and asymptotic infections form the majority of dengue infections in Singapore [33], this is unlikely to lead to inaccurate quantifications of the effect of adult *Aedes* abundance on the risk of dengue transmission as the infection to case notification ratio is unlikely to vary with *Aedes* abundance. The Gravitrap, like all adult traps, catches only a fraction of the mosquito population. Despite that, it allows us to assess the relative abundance of *Aedes* across time and space. Furthermore, our study has shown that the Gravitrap is sensitive enough to indicate transmission risk, demonstrating the relevance of Gravitraps catches. We were unable to account for the effects of vector control activities on dengue transmission. Home inspections and community efforts aimed at removing larval habitats and the use of chemical control to reduce adult mosquito population could to some extent mask the true association between *Aedes* abundance and risk of dengue transmission.

With the disease burden of dengue increasing across the world, identifying specific locations where dengue outbreak risks are the highest is of critical importance [8]. Our study strengthens the evidence for the use of adult *Ae*. *aegypti* indices for dengue risk assessment and early warning for dengue outbreaks. We showed that the adult *Ae*. *aegypti* abundance can allow early identification of geographic localities at high risk of dengue transmission for advance vector control activities. Therefore, the use of adult *Aedes* entomological surveillance in conjunction with a disease surveillance system can benefit dengue control strategies in other cities where dengue is endemic.

## Supporting information

S1 Fig Unmodified boxplot showing the monthly distribution of GIaeg (left) and GIalbo (right). (TIF)

S1 Table Results of univariate analysis. (DOCX)

S2 Table Results of final multivariate regression analysis. (DOCX)
