# Supplementary figures and images for: Short Report: Adult Aedes abundance and risk of dengue transmission
Source: PLoS Negl Trop Dis. 2021 Jun 3;15(6):e0009475. doi: 10.1371/journal.pntd.0009475 (PMC8205144; doi:10.1371/journal.pntd.0009475)

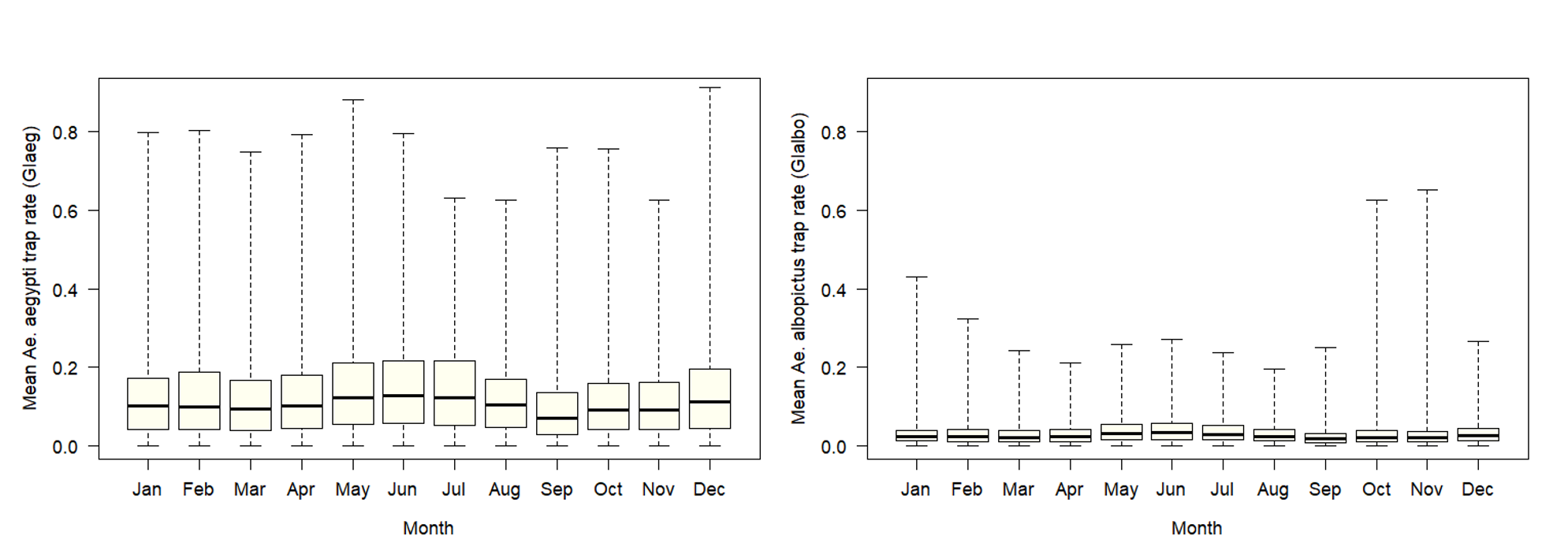

Supplement: S1 Fig — (TIF) [file pntd.0009475.s001.tif]
