# Supplementary material for: Short Report: Adult Aedes abundance and risk of dengue transmission
Source: PLoS Negl Trop Dis. 2021 Jun 3;15(6):e0009475. doi: 10.1371/journal.pntd.0009475 (PMC8205144; doi:10.1371/journal.pntd.0009475)
Supplement: S1 Table — (DOCX) [file pntd.0009475.s002.docx]

S1 Table. Results of univariate analysis

| Variable | | Odds Ratio | 95% CI |
| --- | --- | --- | --- |
| **Mean *Ae. aegypti* trap rate (GI_aeg_)** | |  |  |
| Low: | GI_aeg_ < 0.05 | Referent |  |
| Moderate: | 0.05 ≤ GI_aeg_ < 0.10 | 2.22 | 1.37 – 3.60 |
| High: | 0.10 ≤ GI_aeg_ < 0.17 | 3.30 | 2.07 – 5.27 |
| Very High: | GI_aeg_ ≥ 0.17 | 4.14 | 2.63 – 6.49 |
| **Mean *Ae. albopictus* trap rate (GI_albo_)** | |  |  |
| Low: | GI_albo_ < 0.01 | Referent |  |
| Moderate: | 0.01 ≤ GI_albo_ < 0.02 | 1.58 | 1.04 – 2.40 |
| High: | 0.02 ≤ GI_albo_ < 0.04 | 1.86 | 1.27 – 2.73 |
| Very High: | GI_albo_ ≥ 0.04 | 1.50 | 1.01 – 2.24 |
| **Geographical district** | |  |  |
| Central | | Referent |  |
| North-East | | 2.99 | 2.06 – 4.36 |
| North-West | | 1.73 | 1.15 – 2.60 |
| South-East | | 1.12 | 0.61 – 2.06 |
| South-West | | 1.61 | 1.05 – 2.49 |
| **Epidemiological Year** | |  |  |
| 2017 | | Referent |  |
| 2018 | | 1.65 | 1.14 – 2.40 |
| **Epidemiological Month** | |  |  |
| January | | Referent |  |
| February | | 0.46 | 0.14 – 1.53 |
| March | | 0.46 | 0.22 – 0.99 |
| April | | 0.31 | 0.13 – 0.75 |
| May | | 0.62 | 0.33 – 1.15 |
| June | | 0.73 | 0.40 – 1.32 |
| July | | 0.54 | 0.28 – 1.03 |
| August | | 0.49 | 0.26 – 0.92 |
| September | | 0.62 | 0.31 – 1.22 |
| October | | 0.65 | 0.39 – 1.09 |
| November | | 0.72 | 0.42 – 1.23 |
| December | | 0.98 | 0.61 – 1.58 |
