# Supplementary material for: Short Report: Adult Aedes abundance and risk of dengue transmission
Source: PLoS Negl Trop Dis. 2021 Jun 3;15(6):e0009475. doi: 10.1371/journal.pntd.0009475 (PMC8205144; doi:10.1371/journal.pntd.0009475)
Supplement: S2 Table — (DOCX) [file pntd.0009475.s003.docx]

S2 Table. Results of final multivariable regression analysis

| Variable | | Odds Ratio | 95% CI |
| --- | --- | --- | --- |
| **Mean *Ae. aegypti* trap rate (GI_aeg_)** | |  |  |
| Low: | GI_aeg_ < 0.05 | Referent |  |
| Moderate: | 0.05 ≤ GI_aeg_ < 0.10 | 2.38 | 1.45 – 3.89 |
| High: | 0.10 ≤ GI_aeg_ < 0.17 | 3.40 | 2.09 – 5.52 |
| Very High: | GI_aeg_ ≥ 0.17 | 3.99 | 2.46 – 6.46 |
| **Mean *Ae. albopictus* trap rate (GI_albo_)** | |  |  |
| Low: | GI_albo_ < 0.01 | Referent |  |
| Moderate: | 0.01 ≤ GI_albo_ < 0.02 | 1.23 | 0.81 – 1.88 |
| High: | 0.02 ≤ GI_albo_ < 0.04 | 1.27 | 0.85 – 1.89 |
| Very High: | GI_albo_ ≥ 0.04 | 0.97 | 0.63 – 1.50 |
| **Geographical district** | |  |  |
| Central | | Referent |  |
| North-East | | 2.95 | 2.02 – 4.31 |
| North-West | | 1.81 | 1.20 – 2.72 |
| South-East | | 1.10 | 0.59 – 2.03 |
| South-West | | 1.64 | 1.06 – 2.53 |
| **Epidemiological Year** | |  |  |
| 2017 | | Referent |  |
| 2018 | | 2.05 | 1.35 – 3.11 |
| **Epidemiological Month** | |  |  |
| January | | Referent |  |
| February | | 0.43 | 0.13 – 1.43 |
| March | | 0.44 | 0.20 – 0.93 |
| April | | 0.29 | 0.12 – 0.70 |
| May | | 0.54 | 0.29 – 1.01 |
| June | | 0.63 | 0.34 – 1.14 |
| July | | 0.47 | 0.24 – 0.90 |
| August | | 0.43 | 0.23 – 0.81 |
| September | | 0.55 | 0.28 – 1.10 |
| October | | 0.83 | 0.49 – 1.40 |
| November | | 0.88 | 0.50 – 1.52 |
| December | | 1.15 | 0.70 – 1.88 |
